# MRI‐based radiomic features for identifying recurrent prostate cancer after proton radiation therapy

**DOI:** 10.1002/acm2.14293

**Published:** 2024-02-26

**Authors:** Kazim Z. Gumus, Samuel Serrano Contreras, Mohammed Al‐Toubat, Ira Harmon, Mauricio Hernandez, Savas Ozdemir, Sindhu Kumar, Nurcan Yuruk, Mutlu Mete, K. C. Balaji, Mark Bandyk, Dheeraj R. Gopireddy

**Affiliations:** ^1^ Department of Radiology University of Florida College of Medicine‐Jacksonville Jacksonville Florida USA; ^2^ Department of Urology University of Florida College of Medicine‐Jacksonville Jacksonville Florida USA; ^3^ Center for Data Solutions University of Florida College of Medicine‐Jacksonville Jacksonville Florida USA; ^4^ Department of Computer Science Southern Methodist University Dallas Texas USA; ^5^ Department of Computer Science and Information Systems Texas A&M University‐Commerce Commerce Texas USA

**Keywords:** MRI, prostate cancer, proton therapy, radiomics, recurrence

## Abstract

**Purpose:**

Magnetic Resonance Imaging (MRI) evaluation of recurrent prostate cancer (PCa) following proton beam therapy is challenging due to radiation‐induced tissue changes. This study aimed to evaluate MRI‐based radiomic features so as to identify the recurrent PCa after proton therapy.

**Methods:**

We retrospectively studied 12 patients with biochemical recurrence (BCR) following proton therapy. Two experienced radiologists identified prostate lesions from multi‐parametric MRI (mpMRI) images post‐proton therapy and marked control regions of interest (ROIs) on the contralateral side of the prostate gland. A total of 210 radiomic features were extracted from lesions and control regions on the T2‐weighted (T2WI) and Apparent Diffusion Coefficient (ADC) image series. Recursive Feature Elimination with Cross‐Validation method (RFE‐CV) was used for feature selection. A Multilayer Perceptron (MLP) neural network was developed to classify three classes: cancerous, benign, and healthy tissue. The 12‐core biopsy results were used as the gold standard for the segmentations. The classifier performance was measured using specificity, sensitivity, the area under receiver operating characteristic curve (AUC), and other statistical indicators.

**Results:**

Based on biopsy results, 10 lesions were identified as PCa recurrence while eight lesions were confirmed to be benign. Ten radiomic features (10/210) were selected to build the multi‐class classifier. The radiomics classifier gave an accuracy of 0.83 in identifying cancerous, benign, and healthy tissue with a sensitivity of 0.80 and specificity of 0.85. The model yielded an AUC of 0.87, 95% CI [0.72–1.00] in differentiating cancer from the benign and healthy tissues.

**Conclusions:**

Our proof‐of‐concept study demonstrates the potential of using radiomic features as part of the differential diagnosis of PCa on mpMRI following proton therapy. The results need to be validated in a larger cohort.

## 1 INTRODUCTION

Accurate and early detection of recurrent prostate cancer (PCa) after radiation treatment has critical importance to guide salvage therapy.^1^, ^2^ Currently, multi‐parametric magnetic resonance imaging (mpMRI) remains the chosen diagnostic modality for the localization and characterization of PCa following radiotherapy.^3^ However, the guidelines on how to evaluate mpMRI for recurrent PCa following proton radiation therapy remain unclear.

Proton beam treatment may cause varying biological effects compared to classic photon radiation due to Bragg peak and reduced lateral scattering.^4^ Tissue changes occurring after proton treatment makes MRI evaluation challenging for radiologists. The utility of PI‐RADS scoring in the evaluation of radio‐recurrent tumors following external‐beam radiation and brachytherapy was reported recently.^5^, ^6^ However, the literature on post‐proton radio‐recurrence is scarce.^7^ Assessing biochemical recurrence (BCR) is crucial to improve patient stratification to guide therapy. The evaluation and imaging of recurrent disease following radiation utilizing mpMRI necessitate specific considerations. There are anatomical alterations as fibrosis takes place; the prostate shrinks and different signal patterns emerge on T2‐weighted (T2WI) and the apparent diffusion coefficient (ADC) images. Therefore, outcomes are susceptible to inter‐observer variability.^8^

Recently, radiomics methods have attracted increasing attention in medical image analysis.^9^ Radiomics is the quantification of medical image phenotypes. It extracts numerous mathematical features from medical images that can later be modeled to make classification or prediction with the goal of supporting clinical decision‐making.^10^ Radiomics can quantify the shape, texture, and intensity characteristics of tumors, allowing us to compare these features to the benign tissues such as post‐radiation scarred tissue, prostatitis, and so forth.^11^, ^12^

MRI‐based radiomic approaches were recently reported in several PCa studies including assessment of PCa staging and extraprostatic extension.^10^, ^13^ However, their potential to analyze BCR in PCa following proton therapy has not been studied yet. This study aims to characterize the significant radiomics features and show its feasibility to detect recurrent PCa after proton beam therapy using a deep learning classifier.

## 2 METHODS

Institutional Review Board (IRB) approval was obtained for this study. Patients who underwent mpMRI followed by MRI‐fusion biopsy at our institution from November 2017 to May 2020 were reviewed. The inclusion criteria included patients with histologically confirmed PCa following biochemical recurrence after proton radiation therapy using Phoenix criteria (>PSA nadir+2 ng/mL).^14^ Patients with known metastatic disease were excluded. A total of 12 patients who met all the criteria from our database were selected as the study cohort. All biopsies were performed by fellowship‐trained urologists with the use of the UroNav Machine (Philips Medical Systems, Bothell, WA, USA).^15^

### 2.1 MRI

The patients underwent an mpMRI of the pelvis with and without contrast protocol at our institution using a three Tesla scanner (Trio; Siemens, Erlangen, Germany). The protocol consisted of axial T1‐weighted (T1WI) and high‐resolution T2WI with more than two planes of multiplane (axial, sagittal, or coronal) and without fat suppression. Diffusion Weighted Imaging (DWI) was performed during free breathing with axial plane water‐excited fat‐suppressed single‐shot spin echo‐planar sequence. Dynamic Contrast Enhanced (DCE) MRI was done using T1‐weighted fat‐suppressed sequences before and after IV injection of contrast (DOTAREM, Bayer Pharma, Germany) at a dose of 0.1 mm/kg at a rate of 2 mL/s. Dynamic images were obtained at 20 s (arterial phase), followed by 70 s (venous phase), and then delayed phase at 3 and 10 min. The MRI sequence parameters for T2WI and DWI are given in Table 1. Two fellowship‐trained radiologists, blinded to biopsy results, reviewed the mpMRI images in consensus. They segmented a total of 18 lesions on each slice of the T2WI and ADC images using itk‐SNAP 3.8.0 software.^16^ For each lesion contouring, they also segmented a control region of interest (total 18) of the approximately same size and shape on the contralateral side of the prostate gland which was considered as healthy in this study.

**TABLE 1:** MRI sequence parameters for the T2WI and DWI.

	T2WI	DWI
Repetition Time (TR) (ms)	5870	4500
Echo Time (TE) (ms)	96	84
FOV (mm)	232 × 232	221 × 260
Matrix	320 × 320	136 × 160
Slice Thickness (mm)	3	4
Slice Spacing (mm)	3	5
*b*‐value	–	0‐500‐1500

### 2.2 Radiomics analysis

We normalized the signal intensities on the T2WI to eliminate scan‐to‐scan variations.^17^ No normalization was performed on the ADC images since ADC reflects the quantitative measure of physiological processes.^18^ We performed in‐plane resampling on both the T2‐weighted (0.5 × 0.5 mm) and ADC images (1.5 × 1.5 mm) to homogenize image processing.^19^

We used the PyRadiomics package to generate radiomics features.^20^ PyRadiomics is an open source radiomics package developed by van Griethuysen et al.^20^ It divides image processing into four steps: preprocessing, filtering, feature extraction, and reporting. Most of the features are in line with the Imaging Biomarker Standardization Initiative (IBSI).^21^ We extracted 105 radiomic features from each imaging sequence: the T2‐weighted and ADC images. Radiomics features from the following feature classes were included: First Order (*n* = 18), Shape (*n* = 14), and texture (*n* = 73) including Grey Level Co‐occurrence Matrix (GLCM, *n* = 22), Grey Level Size Zone Matrix (GLSZM, *n* = 16), Grey Level Run Length Matrix (GLRLM, *n* = 16), Grey Level Dependence Matrix (GLDM, *n* = 14), and Neighboring Gray Tone Difference Matrix (NGTDM, *n* = 5). A complete list of radiomics features is given in the Reference.^20^

### 2.3 Feature selection

After combining the ADC and T2 features of each subject in a feature vector, we used the Recursive Feature Elimination with Cross‐Validation (RFE‐CV),^22^ which is originally based on Support Vector Machines (SVM).^23^ RFE‐CV is available in the Scikit‐learn library for feature selection.^24^ It is an iterative approach to selecting a subset of features by recursively eliminating less important features and simultaneously ensuring optimal performance through cross‐validation. RFE‐CV is conducted initially prior to executing a comprehensive LOO validation. The result is a ranking of features and a set of optimal features that empirically maximize the prediction accuracy for the underlying classification problem. In this study, all 210 features per lesion were sorted using RFECV with a logistic regression estimator instead of linear SVM. Within RFE‐CV, features are ranked by the absolute value of the coefficients assigned to each feature. The rationale is that a larger absolute coefficient value indicates a stronger effect on the dependent variable. The ranked features enabled us to design a classifier with the *N* most informative features to detect PCa recurrence.

### 2.4 Classifier design

In preprocessing step, each feature was scaled between −1 and 1. Feature scaling around 0 (min‐max or standardization) can substantially speed up the Multilayer Perceptron (MLP) training.^25^ We used a multiclass Multilayer Perceptron (MLP)^25^ neural network as the primary classifier for this study, which was trained with backpropagation algorithm. We chose the MLP as a classifier because MLPs excel at capturing intricate interactions between various radiomic features, which can hold valuable information for diagnosis or prognosis. This is particularly beneficial when dealing with large datasets containing diverse features, as MLPs can leverage these interactions for more accurate predictions. The MLP architecture consisted of five hidden layers of 64, 32, 16, 9, and 6 neurons, respectively. The leaky rectified linear unit (ReLU) activation function was employed with an alpha value of .1. Leaky ReLU allows a small gradient when the unit is not active (output less than 0), which helps mitigate the vanishing gradient problem. The output layer had three units and used the SoftMax activation function. This was the final layer responsible for producing the model's output probabilities for the three classes, such as [0.25 0.55 0.20]. Each model was trained with 100 epochs and controlled with early stopping monitor (*s* = 15).

### 2.5 Cross‐validation

We used Leave‐One‐Out (LOO) cross‐validation method to validate our model. In LOO cross‐validation, a single observation from the dataset is used as the validation set while the remaining observations (35 images) form the training set. This process was repeated until every observation in the dataset has been used as the validation set once. Note that LOO cross‐validation with 10 features for 36 regions of interest took around 40 s on the regular desktop computer (3.5 MHz Intel i7, 12GB, Scikit‐learn v. 1.3.221).

### 2.6 Evaluation of classification model

The confusion matrix was used to determine instances in a predicted class (cancerous, benign, and healthy) while each row displayed instances in a true class. Sensitivity and specificity values for the radiomics classifier were calculated. The AUC of the receiver operating characteristics curve for the cancer‐vs‐rest and the 95% confidence interval (CI) were calculated.

## 3 RESULTS

**FIGURE 1:**
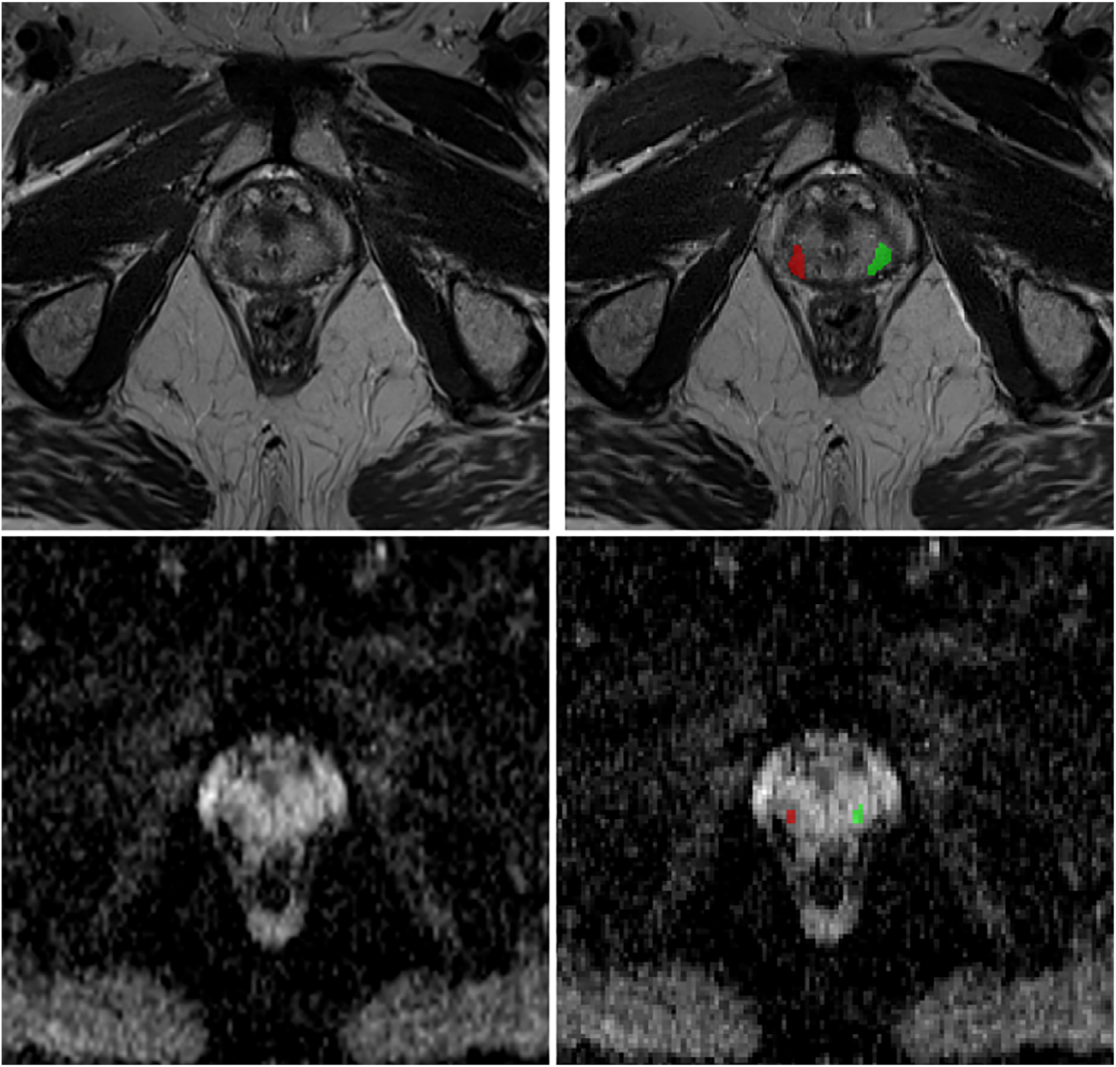
T2‐weighted (top) and ADC (bottom) images of a recurrent PCa patient as an illustration. The right images show the segmentations: tumor (red) and contralateral control (green).

Of the 12 patients with biochemical recurrence, MRI‐guided fusion biopsy identified seven patients (7/12) with recurrent PCa post proton therapy (10 lesions). However, MRI‐guided biopsy found five patients (5/12) with benign lesions (eight lesions). Figure 1 shows the MRI images of a patient with recurrent PCa and the segmentations made by the radiologists as an example.

From a total of 210 quantitative features, 10 key radiomic features were selected utilizing the RFE‐CV algorithm (Table 2). Five features (5/10) were derived from the ADC images and five features (5/10) were derived from the T2‐weighted images. The majority of the ADC features were first‐order (3/10) while the remaining features were texture‐related (1/5) and shape‐related (1/5). Among the T2WI features, three (3/5) were texture‐related, the others were shape‐ (1/5) and intensity‐related (1/5) (Table 2).

**TABLE 2:** A heat map describing the importance of 10 selected features of multi‐class radiomic model for PCa recurrence classification.

Rank	Radiomic feature
52.0	T2 GLCM Difference Variance
35.3	ADC First Order Minimum
20.9	T2 GLDM Large Dependence Low Gray Level Emphasis
19.2	T2 First Order 10 Percentile
18.5	ADC First Order Interquartile Range
13.9	ADC Shape Maximum 3D Diameter
13.1	ADC First Order Maximum
8.2	ADC NGTDM Coarseness
6.9	T2 GLSZM Zone Variance
4.8	T2 Shape Least Axis Length

Abbreviations: GLCM: Gray‐Level Co‐Occurrence Matrix, GLDM: Gray‐Level Dependence Matrix, GLSZM: Gray‐Level Size Zone Matrix; NGTDM: Neighboring Gray Tone Difference Matrix.

**FIGURE 2:**
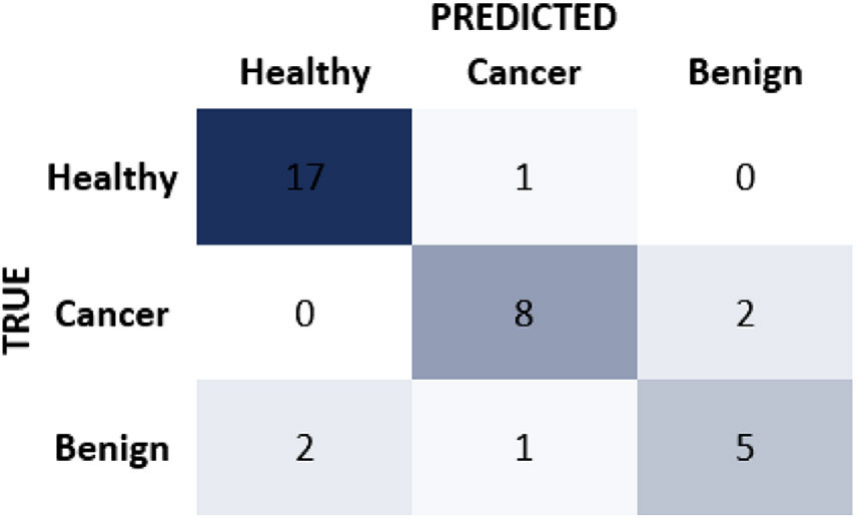
Confusion matrix of the MLP classifier.

**FIGURE 3:**
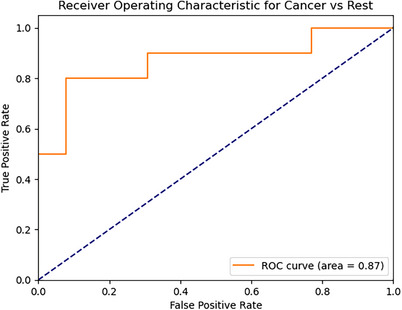
Cancer‐vs‐Rest ROC curve.

**FIGURE 4:**
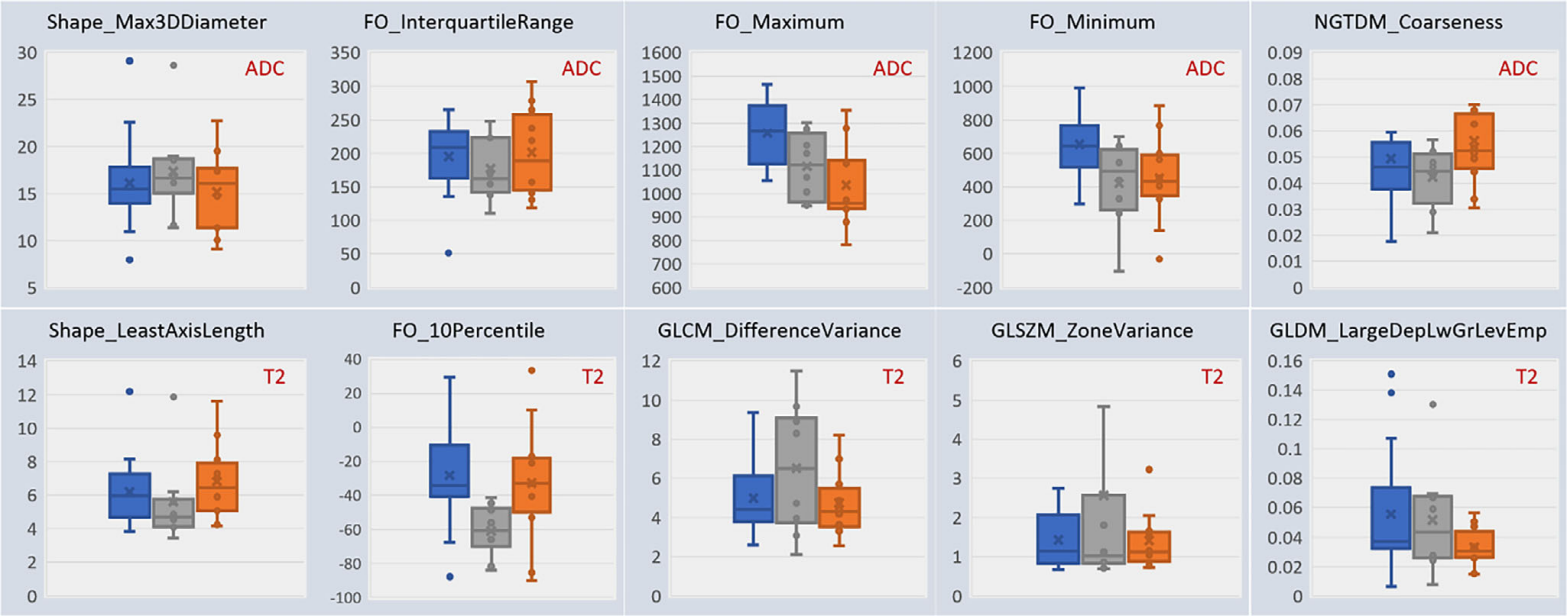
Box plot showing 10 selected radiomic features across cancer (orange), benign (gray), and healthy (blue) tissue. GLCM, Gray‐Level Co‐Occurrence Matrix; GLDM, Gray‐Level Dependence Matrix; NGTDM, Neighboring Gray Tone Difference Matrix; GLSZM, Gray‐Level Size Zone Matrix; FO, First Order; LargeDepLwGrLevEmp, Large Dependence Low Gray Level Emphasis.

Figure 2 shows the confusion matrix. The multi‐class radiomics classifier gave an accuracy of 0.83 in identifying cancerous, benign or healthy tissues with a sensitivity of 0.80 and specificity of 0.85. The model gave an AUC of 0.87 in differentiating cancer from other tissues including healthy and benign (Figure 3). The data characteristics of 10 radiomic features across cancerous, benign, and healthy tissue are displayed in a box figure in Figure 4.

## 4 DISCUSSION

The biologic and radiologic effects of proton therapy on prostate cancer tissue are complex as high doses of proton particles could cause unpredictable changes in tissue characteristics, making the interpretation of MRI images highly challenging for radiologists. Radiomics could play a supportive role in analyzing these images beyond the analysis of naked eyes. This study examines the key radiomic features and demonstrates the promising role of MRI‐based radiomics for improved identification of recurrent PCa after proton therapy.

We found a total of 10 radiomic features: half of them from the T2‐weighted images and the other half from the ADC images. The T2WI features were mostly texture‐related while the ADC features were mostly first‐order intensity‐related. While the texture features describe the complexity and coarseness of the ROI, first‐order features provide information about the distribution of voxel intensities within the ROI. On the ADC images, the mean values of the maximum 3D diameter, first order maximum, and first order minimum values were lower in cancerous lesions while the mean values of the first order interquartile range and NGTDM coarseness were higher (Figure 4). On the T2WI, the mean values of the least axis length and first order 10 percentile were higher in cancerous lesions while the GLCM difference variance, GLSZM zone variance, and GLDM large dependence low gray level emphasis values were lower. It can be concluded that post‐radiation recurrent PCa displays measurable first‐order and texture characteristics on the MRI images. We suggest that these features to be further explored in untreated prostate cancer to assess the specificity of these changes to proton therapy.

We used the 10 radiomic features in building a multi‐class MLP model. The model provided high accuracy (0.83) in differentiating the recurrent PCa from radiation‐induced benign changes and normal appearing healthy tissue with high sensitivity and specificity. Due to the small sample size, we used the LOO algorithm to validate our model. LOO is a robust cross‐validation method, well‐suited for small‐sample classification problems. Given the nature of LOO where each data point is used for validation exactly once, it offers a low bias since it allows for a larger and more representative training set. It is particularly advantageous in situations with limited data because it maximizes the training data available. Although the main drawback is its computational intensity, its benefits often outweigh its costs, especially in studies with small sample sizes where accurate generalization is crucial.

After examining the confusion matrix and ROC curve, it can be inferred that the neural network classifier effectively captures the relationships between classes and features. The classifier correctly identifies 8 out of 10 cancer lesions. The missed two lesions were identified as benign lesions rather than normal appearing heathy tissue. The classifier also correctly detected five out of eight benign lesions. Among the missed three lesions, only one of them was identified as cancer. These results indicate that the classifier has promising sensitivity and specificity.

To our knowledge, this is the first study to analyze and assess the radiomic features of recurrent PCa on mpMRI images after proton therapy. Recently, Bazargani et al. reported that diffusion heterogeneity had the strongest association with recurrence of PCa post proton therapy (7). Our study corroborates this finding at the level of radiomics. We observed that ADC as well as T2WI had provided radiomic features that could be used in differential diagnosis of recurrent PCa cancer (Table 2).

Several studies have demonstrated a high degree correlation between PI‐RADS classification and the detection of clinically significant PCa. Despite correlation between PI‐RADS scoring and presence of clinically significant PCa, 40% and 17% of PI‐RADS 4 and 5 lesions do not have cancer, respectively.^26^ Radiomic features have been shown to enhance the accuracy of PI‐RADS system.^7^ While most of these were done on treatment‐naïve prostate data, detecting PCa following proton therapy is sparse.^13^, ^27^, ^28^ While PI‐RADS demonstrates high correlation between readers, there is still a substantial discordance rate based on imaging features and levels of expertise.^29^ Therefore, we believe that radiomics could be a promising and exceptionally reliable tool that supports clinical decision‐making with less mobility for the patient.

There are several limitations to this study. First, the total sample size was small. We note that the results presented are from a pilot study. Second, the datasets were acquired from only three Tesla scanners at a single institution. This limits the generalizability of the reported findings to other magnets. Third, this study assessed radiomic features extracted from only T2‐weighted and ADC images. Hence, the radiomic value of DWI and T1‐weighted DCE images in this setting needs further investigation.

## 5 CONCLUSION

In conclusion, this proof‐of‐concept study demonstrates that MRI‐based radiomic features could assist with improved detection of recurrent PCa after proton therapy. The findings need further validation on large cohorts.

## AUTHOR CONTRIBUTIONS

All authors contributed to this paper with the conception and design of the study, literature review and analysis, drafting and critical revision and editing, and final approval of the final version.

## CONFLICT OF INTEREST STATEMENT

Authors declare no conflict of interests for this article.
